# Supplementary material for: Effects of Arthrospira platensis Extract on Physiology and Berry Traits in Vitis vinifera
Source: Plants (Basel). 2020 Dec 19;9(12):1805. doi: 10.3390/plants9121805 (PMC7766242; doi:10.3390/plants9121805)
Supplement: Supplementary file 1 [file plants-09-01805-s001.zip › Supp Figure 1.docx]

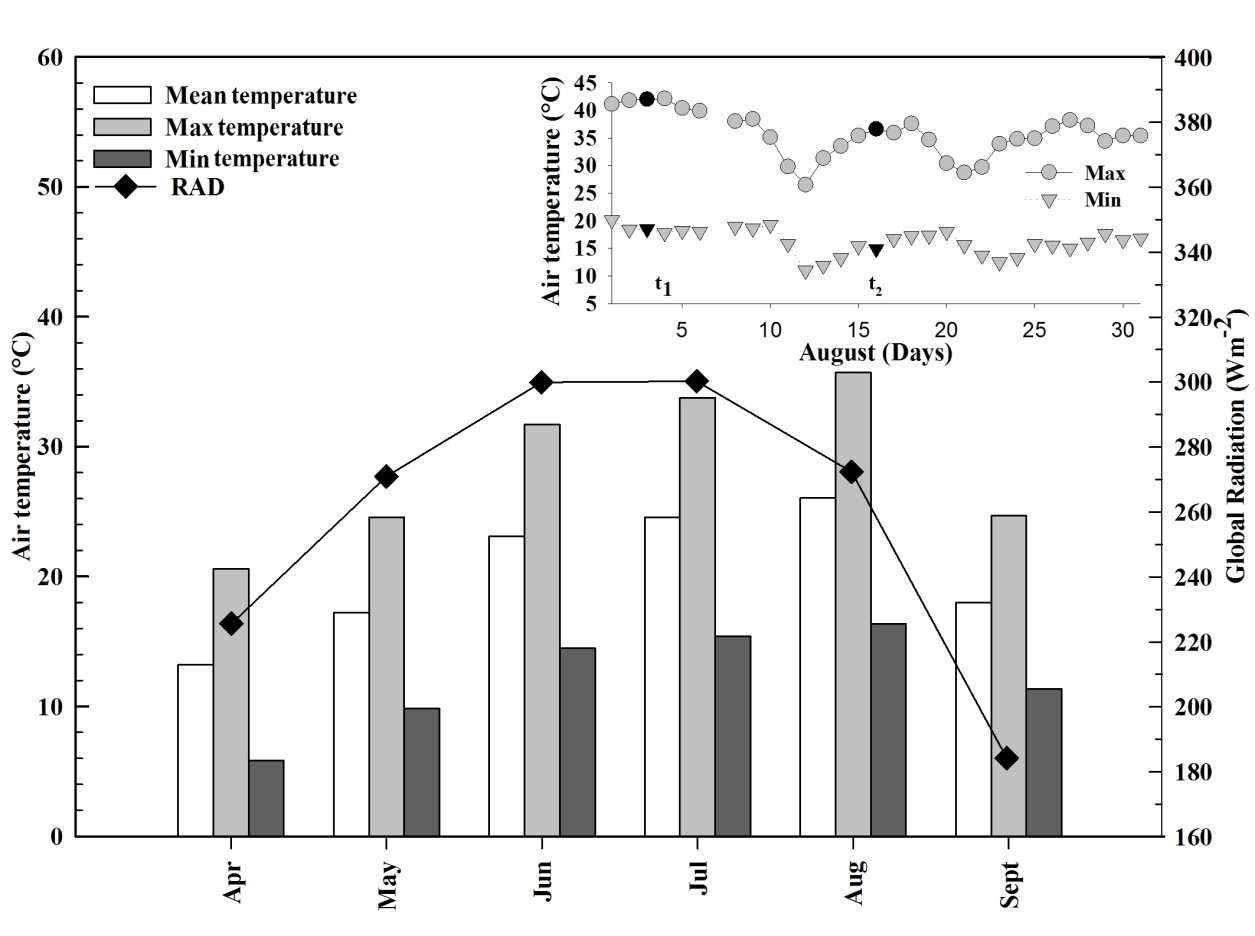


**Supplementary Figure 1. Weather conditions of the experimental site.** Monthly averages of mean (white bars), maximum (grey bars) and minimum (dark grey bars) air temperatures (°C) and global radiation (RAD, W m^-2^) of the growing season; the inset show the daily averages of maximum (grey circles) and minimum (grey triangles) air temperatures (°C) measured in August. In the inset, black circles and triangles represent maximum and minimum air temperatures at t_1_ and t_2_. This figure was yet published in [39].
